# High precise dating on the variation of the Asian summer monsoon since 37 ka BP

**DOI:** 10.1038/s41598-021-88597-7

**Published:** 2021-04-30

**Authors:** Ting-Yong Li, Yao Wu, Chuan-Chou Shen, Jun-Yun Li, Hong-Wei Chiang, Ke Lin, Liang-Cheng Tan, Xiu-Yang Jiang, Hai Cheng, R. Lawrence Edwards

**Affiliations:** 1 Yunnan Key Laboratory of Plateau Geographical Processes and Environmental Change, Faculty of Geography, Yunnan Normal University, Kunming, 650500 China; 2 Chongqing Key Laboratory of Karst Environment, School of Geographical Sciences, Southwest University, Chongqing, 400715 China; 3 High-Precision Mass Spectrometry and Environment Change Laboratory (HISPEC), Department of Geosciences, National Taiwan University, Taipei, 10617 Taiwan; 4 State Key Laboratory of Loess and Quaternary Geology, Institute of Earth Environment, Chinese Academy of Sciences, Xi’an, 710075 China; 5 College of Geographical Science, Fujian Normal University, Fuzhou, 350007 China; 6 Institute of Global Environmental Change, Xi’an Jiaotong University, Xi’an, 710049 China; 7 Department of Earth and Environmental Sciences, University of Minnesota, Minneapolis, MN 55455 USA; 8 Department of Geosciences, National Taiwan University, Taipei, 10617 Taiwan, ROC; 9 Present Address: Earth Observatory of Singapore, Nanyang Technological University, Singapore, 639798 Singapore

**Keywords:** Climate sciences, Environmental sciences, Planetary science

## Abstract

Comprehensive comparison of paleoclimate change based on records constrained by precise chronology and high-resolution is essential to explore the correlation and interaction within earth climate systems. Here, we propose a new stalagmite-based multidecadal resolved Asian summer monsoon (ASM) record spanning the past thirty-seven thousand years (ka BP, before ad 1950) from Furong Cave, southwestern China. This record is consistent with the published Chinese stalagmite sequences and shows that the dominant controls of the ASM dynamics include not only insolation and solar activity but also suborbital-scale hydroclimate events in the high latitudes of the northern hemisphere, such as the Heinrich events, Bølling-Allerød (BA), and Younger Dryas (YD). Benefit from the unprecedented accurate chronology, the timings of these events are precisely dated, with uncertainties of, at most, 40 years (2σ). The onset of the weak ASM during the YD began at 12.92 ka BP and lasted for 430 years. The occurrence of the 200-yr Older Dryas during the BA period was dated from 13.87 to 14.06 ka BP. The durations of the three Heinrich (H) events, H1, H2, and H3, are 14.33–18.29, 23.77–24.48, and 28.98–30.46 ka BP, respectively. Furong record shows surprisingly variable onset transitions of 980, 210, and 40 years for the corresponding weak ASM events. These discrepancies suggest different influences of the H events on ASM dynamics. During the periods of H 1–3, the obvious difference between our Furong record and NGRIP δ^18^O record indicated the decoupling correlation between the mid-low latitudes and high latitudes. On the other hand, synchronous climate change in high and low latitudes suggests another possibility which different to the dominant role of Northern high latitudes in triggering global climate change. Our high quality records also indicate a plausible different correlation between the high and mid-low latitudes under glacial and inter-glacial background, especially for the ASM regimes.

## Introduction

Over the past eight hundred thousand years, the orbital-scale glacial-interglacial cycles were punctuated with suborbital dynamics (EPICA, 2004). The internal hydroclimate fluctuations of the last glacial period (LGP) have remained the most studied such phenomena of the past decades^1,2^. One of the remarkable features of this period is a series of abrupt events in the northern hemisphere (NH)^3^.

During the last deglaciation, influxes of fresh water from Lake Agassiz^4^ that cascaded into the North Atlantic decreased the salinity of surface water, weakened the Atlantic Meridional Overturning Circulation (AMOC), changed the thermohaline circulation (THC), and influenced the global climate^5^. These centurial-to-millennial scale climatic oscillations correspond to the Older Dryas (OD)^6^, Younger Dryas (YD)^7^, and 8.2 ka event^8^, all of which are associated with a decline of the intensity of the Asian summer monsoon (ASM)^9^ through ocean–atmosphere coupling processes. Low-latitude monsoonal hydroclimatic responses are apparently slower than the transitions of the onsets and terminations of these high-latitude abrupt climate events^10^. Partin et al.^11^ used stalagmite records in the Philippines and eastern and southern China to propose that changes in the AMOC may not produce the same effects across the entire AM territory. The robustness of this argument should be verified with additional regional ASM records.

During the last glaciation, enormous icebergs intermittently calved from the ice shelves and traversed the North Atlantic. These so-called Heinrich (H) events were recorded at least six times through the ice-rafted debris (IRD) preserved in marine sediment^2,12^. During H events, the extensive amounts of fresh water entering the North Atlantic attenuated the density-driven THC and led to regional climate changes worldwide^2^. ASM intensity was also reduced^9,13,14^. Due to chronology limitations and proxy resolutions, the detailed transitions and dynamics of ASMs during H events have never been precisely dated. There are different mechanisms in the response of high and low latitudes climate variations to series H events^15–19^. Climate and environment changes at high and low latitudes can be identified from multiple climate proxy records (e.g. δ^18^O, ^17^O-excess, d-excess) in the Greenland ice core (NGRIP^15,17,20,21^. During the H1 and H4, although the temperature in Greenland maintained a stable low temperature, large-scale climate reorganization related to warming and humidification in the low latitude areas^15–17^. These observations argued the traditional opinion that the dynamics of ASM were deeply dominated by climate changes in the north high latitudes^9^. It is still an open question whether this decoupling between high latitudes and mid-low latitudes existing during all the H events in the last glacial period (LGP).

Here, we present a 37-kyr stalagmite oxygen isotope record from southwestern China. For this stalagmite, the section of 6–16 ka BP had been reported by Li et al.^22^. This work reports the complete record of this stalagmite. The YD, OD, and H events are precisely dated, with only decadal-scale errors of just a few decades. The detailed structures of these events would provide a better understanding of low-latitude ASM responses through the internal forcings that originated from the NH high latitudes as well as allowing an evaluation of the fidelity of the Greenland ice core chronologies.

## Regional settings and samples

**Figure 1:**
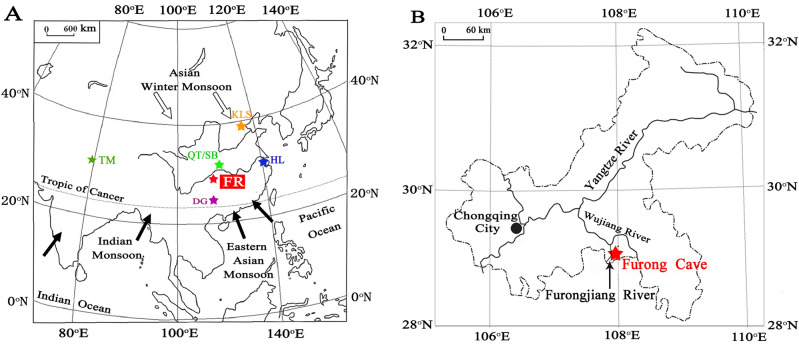
(**A**) Locations of Furong (this study) and other Chinese caves. Solid stars denote the cave sites of the Furong (red), Dongge (purple)^30^, Hulu (blue)^9^, Qingtian and Sanbao (QT/SB) (green)^29,48^, Kulishu (dark yellow)^49^, and Timta caves (dark green)^47^. (**B**) A map of Chongqing municipal city. Furong Cave (red star) is located near the Furongjiang River and is 5 km from the Wujiang River^22^.

Furong Cave (29°13′44″ N, 107°54′13″ E) is located in the Cambrian limestone and dolomite strata of the eastern bank of the Furongjiang River, which is a secondary branch of the Yangtze River, Chongqing, southwestern China (Fig. 1). The overlying vadose zone is 300–500 m thick, the elevation at the cave entrance is 480 m above sea level, the cave air temperature is 16.3 °C and the humidity is 95–100%. Furthermore, the regional climate is dominated by the Asian monsoon system, characterized by cold-dry winters and hot-wet summers. The current annual average precipitation is 1200 mm, with 70% of the annual rainfall occurring during the wet season from May to October. Details of the geographic and regional hydrological settings of the cave were described in Li et al.^22^.

**Figure 2:**
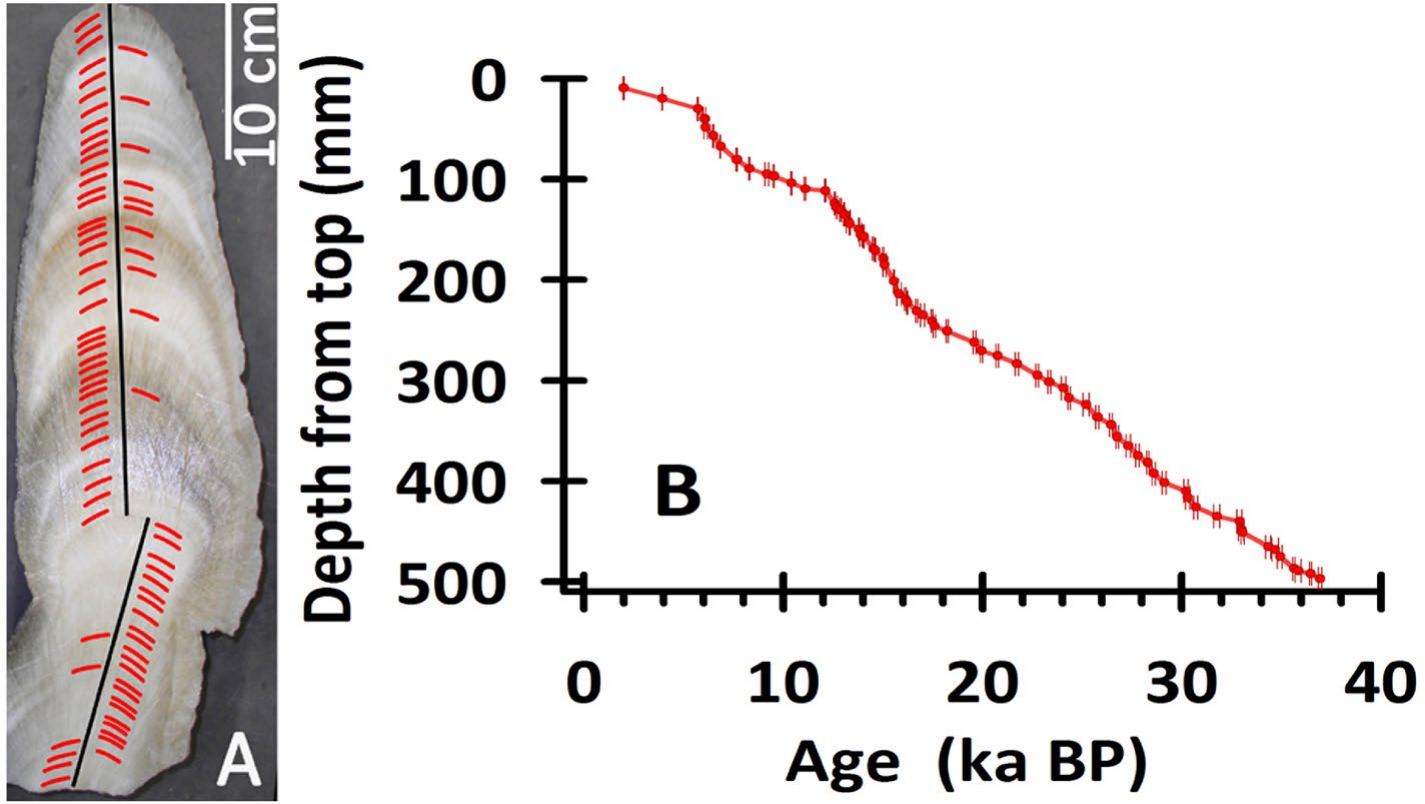
(**A**) A photograph of stalagmite FR5 from Furong Cave. The black line shows the track of the stable C/O isotopic analysis. Red markers denote the subsamples drilled for ^230^Th dating. (**B**) An established age model (red line) of 67 ^230^Th dates (red circles) with a 2-sigma uncertainty (Appendix Table 1).

FR5 is an active stalagmite with a total length of 50 cm and diameters vary from 6 cm at its top to 14 cm at its bottom (Fig. 2A). The stalagmite was collected from a chamber 1 km from the natural cave entrance, halved along its growth axis and polished (Fig. 2A). X-ray diffraction and electron backscatter diffraction techniques show that the stalagmite is 100% aragonite, demonstrating the total absence of aragonite-to-calcite transformation^22^.

## Methods

### ^230^Th dating and stable oxygen isotope measurements

A total of 67 layers (Fig. 2A, Appendix Table 1) of 30–180 mg each were drilled in the polished surface of the halved stalagmite for ^230^Th dating using a multicollector inductively coupled plasma mass spectrometer (MC-ICP-MS), Thermo Electron NEPTUNE, at the High-Precision Mass Spectrometry and Environment Change Laboratory (HISPEC), National Taiwan University^23^. The details of the chemistry, instrumental analysis, and off-line calculation are described in Shen et al.^23^. The half-lives of U-Th nuclides used are given in Cheng et al.^24^. The uncertainties in the U-Th isotopic data and ^230^Th dates (ka BP, before 1950 ad) are calculated at the 2σ level or at two standard deviations of the mean (2σ_m_) unless otherwise noted.

For the stable oxygen isotope analysis, 956 subsamples of 50–100 µg each were drilled from along the growth axis using a 0.5-mm carbide dental bit (Fig. 2A). The measurements were taken using an isotope ratio mass spectrometer, specifically the Finnegan DELTA V PLUS coupled with a Kiel IV automated carbonate device at the Laboratory of Geochemistry and Isotopes, Southwest University, China. Measurements of an international standard, NBS 19, and an in-house standard, NCKU 1, show a one-sigma reproducibility of ± 0.10‰ for δ^18^O. All the stalagmite oxygen stable isotopic data are reported in permil (‰) relative to the Vienna Pee Dee Belemnite (V-PDB) standard.

### Identification of climate events

**Figure 3:**
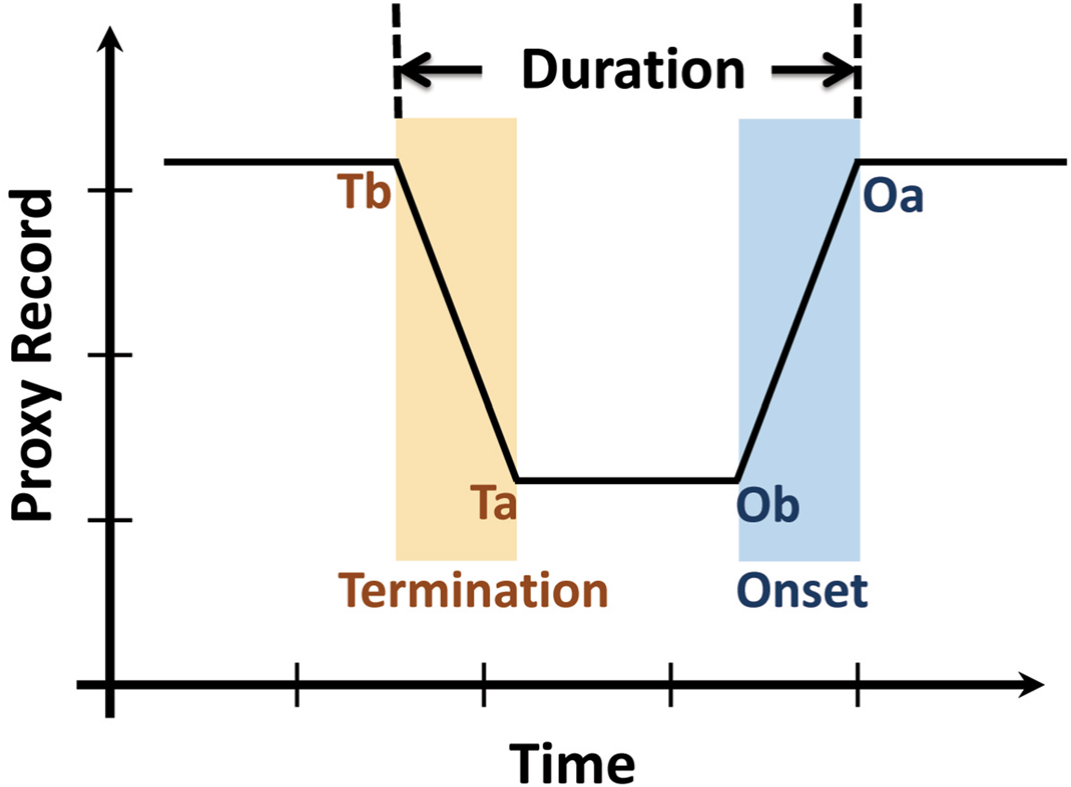
Concept sketch of a proxy-inferred climate event. Blue and yellow shadows denote the transitions of the onset and termination of an event, respectively. See text for details.

Here, we describe our method of defining the periods of climatic events (Fig. 3). Taking event in Fig. 3 as an example, the onset transition is from the inflection point **Oa** to that of **Ob**; similarly, the termination transition is from **Ta** to **Tb**. The period between **Oa** and **Tb,** represents the duration of the event. A statistical regression approach, RAMPFIT^25^, was used to determine the end of the Holocene optimum period (HOP) and the transitions of the YD and H1-H3 events in the FR5 δ^18^O and other proxy records. This weighted least-squares method, which determines the ramp between states of a certain record, has been successfully applied to geochemical proxy time series^26–28^.

## Results

### Chronology

The uranium–thorium content, isotopic compositions and determined ^230^Th ages are given in Appendix Table 1. The ^238^U content of the FR5 dating subsamples varies from 200 to 800 ppb at depths of 0–110 mm to 1000–8000 ppb at depths > 110 mm. For most of the dating subsamples (63/67), the low ^232^Th levels are < 1000 ppt. The age corrections for the initial ^230^Th are only 0–15 years, smaller than dating errors of ± 16–168 years. The age uncertainties of most of the subsamples (63/67) are < 7‰. The ^230^Th ages, ranging from 1.99 to 36.94 ka BP (Appendix Table 1), are in stratigraphic order. An age model is constructed by linearly interpolating between the ^230^Th dates (Fig. 2B).

**Table 1:** Comparison of the timing for some tie-points between the NGRIP (Events in NGRIP determined with ice core δ^18^O record) and Chinese stalagmite records.

Events (or tie point)	Recommendations in NGRIP	Max.count error	References	ASM records	Age (years BP)	Age error (years)	Time difference between	References
	GICC05 age (calibrated to years BP)	(MCE) (years)					NGRIP and ASM records (years)	
8.2 ka BP event (end)	8090	45	^53^	Central China	8100	100	− 10	^40^
8.2 ka BP event (onset)	8250	49	^53^	Central China	8250	100	0	^40^
Start of Holocene	11,601	99	^59^	Northen China	11,540	40	61	^42^
Peak of Late Allerød	12,840	138	^59^	Southwest China	12,877	40	− 37	This study
Older Dryas	13,980	169	^59^	Southwest China	13,985	40	− 5	This study
Start of Bølling	14,635	186	^59^	Southwest China	14,570	40	65	This study

### δ^18^O records

**Figure 4:**
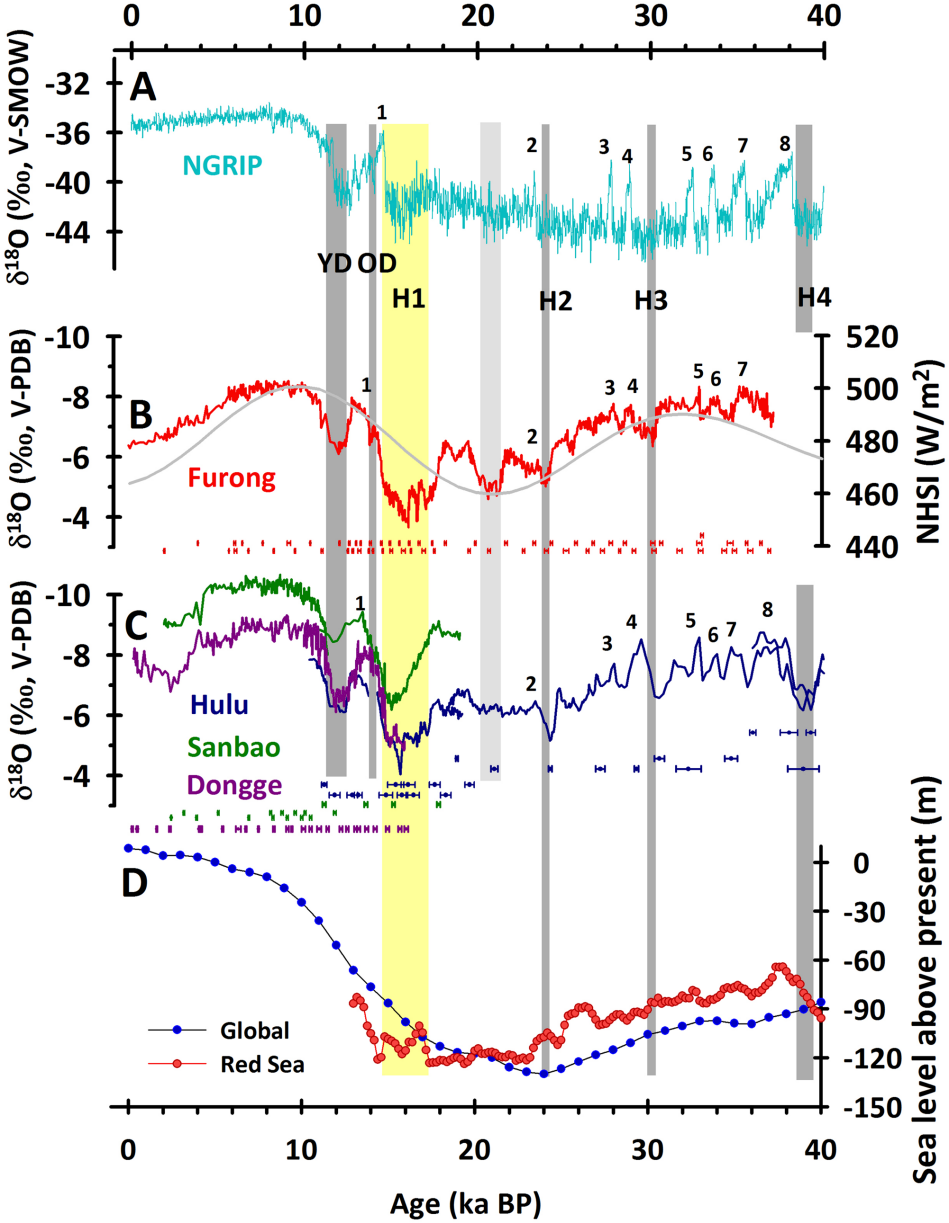
Proxy records from Greenland, Chinese caves, and oceans. (**A**) δ^18^O records of the Greenland ice core, NGRIP^46,55^. Cave stalagmite δ^18^O records of the (**B**) Furong (this study), (**C**) Hulu (blue)^9^, Sanbao (green)^29^, and Dongge (purple) caves^30^. ^230^Th ages and errors are color-coded below the stalagmite records. (**D**) Global ^64^ (blue) and Red Sea^65^ (red) sea level records. The Arabic numerals 1–8 represent the Greenland Interstadials (GIS). Younger Dryas (YD), Older Dryas (OD), and Heinrich events 2–4 (H2–H4), are shown; the gray columns denote the YD, OD, and H2–H4, respectively. H1 is highlighted by a yellow column. The light gray column shows a δ^18^O excursion in FR5 records, which is not seen in the Hulu record. The gray curve denotes the northern hemisphere summer insolation (NHSI) at 30° N (W/m^2^)^66^.

The Furong stalagmite FR5 δ^18^O data range from − 3.65‰ to − 8.53‰, with an averaged temporal resolution of 40 years, and are plotted in Fig. 4B. The δ^18^O data increase gradually from − 8.0‰ at 36.94 ka BP to a maximum of − 3.65‰ at 16.14 ka BP, characterized by several centennial to millennial fluctuations (Fig. 4B). The subsequent δ^18^O data has an enrichment of 2‰ in δ^18^O from 12.89 to 12.11 ka BP, decreasing to a minimum of − 8.53‰ at 9.20 ka BP, followed by a millennially increasing trend rising to − 6.59‰ at 1.99 ka BP.

## Discussion

### Comparison within monsoon China

Previous multiyear in situ monitoring programs and Hendy test results demonstrated that both of the speleothems in Furong Cave and stalagmite FR5 are deposited at oxygen isotopic equilibrium condition and inherited the isotopic composition of precipitation^22^. Strong correlations among contemporaneous sequences are observed in the Furong, Hulu^9^, Sanbao^29^ and Dongge^30^ records from the last glacial period to the Holocene (Fig. 4B,C), supporting previous arguments for stalagmite δ^18^O records mainly capturing monsoonal hydroclimate variability, qualitatively representing ASM intensities (e.g.,^31,32^, and changes in moisture sources which dominated by atmosphere circulations^33–35^.

**Figure 5:**
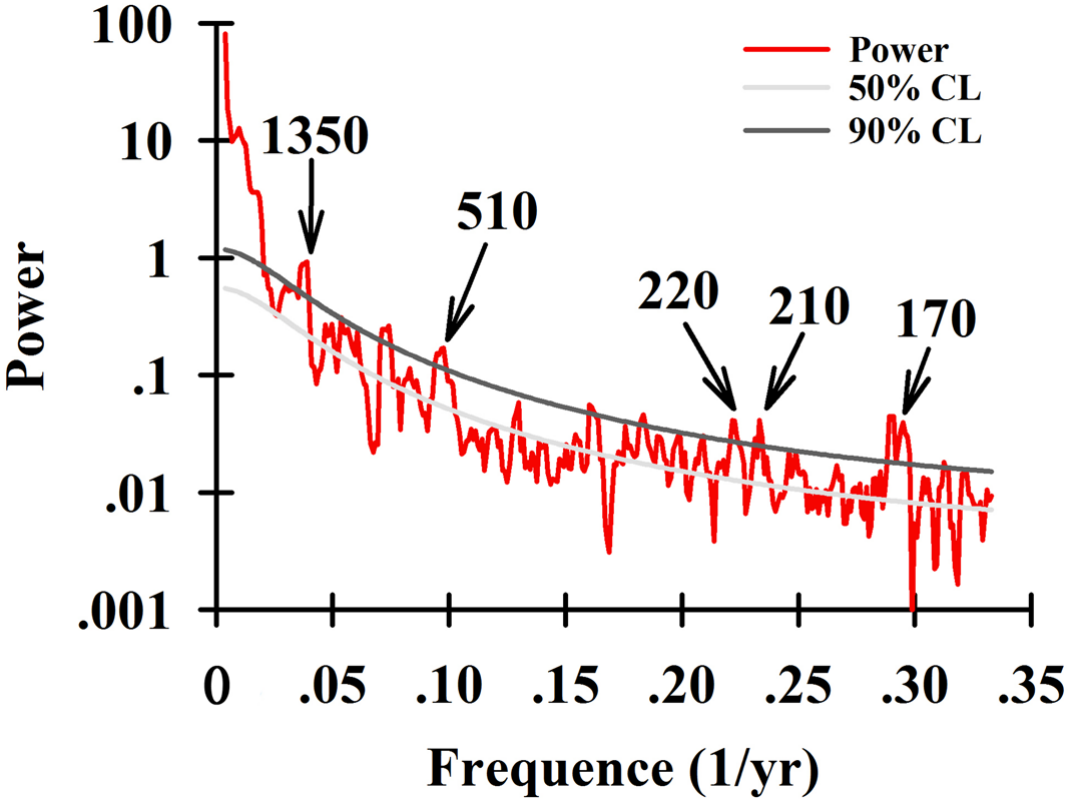
Periodicities of the FR5 δ^18^O records from the multi-taper method (MTM)^36^. The red curve is the spectral power. The dark and light gray lines denote the confidence levels (CL) of 90% and 50%, respectively. The prominent periods of 170 year, 210 year, 220 year, 510 year and 1350 year are highlighted.

As the almost only source of energy for earth climate systems, solar activity dominates the energy input into earth surface. The tropical Indian Ocean and Pacific Ocean are the heat and vapor sources of ASM. Variations in energy input and distribution in earth surface influence on the thermodynamic gradient between continents and oceans, resulting the changes in ASM intensity, modulating the portion of summer precipitation (lighter δ^18^O values than that in winter precipitation), and/or changing the distance of moisture source via the re-organization of atmospheric circulations which may be regulated by ENSO^34,35^ (see details in “Asynchronous regional Holocene ASM”). All these physical processes influenced on the precipitation δ^18^O and were finally reserved in speleothems. Multi-millennial-scale trends of FR5 δ^18^O-inferred ASM variability show a clear correlation with the precession-dominated NH solar insolation (NHSI) (Fig. 4). Series significant periodicities of 170, 210, 220, 510 and 1350 years (Fig. 5), produced by a spectral analysis method^36^, support that the solar activity plays an essential role in ASM variability on the centennial to millennial timescales^9,32^.

### Asynchronous regional Holocene ASM

**Figure 6:**
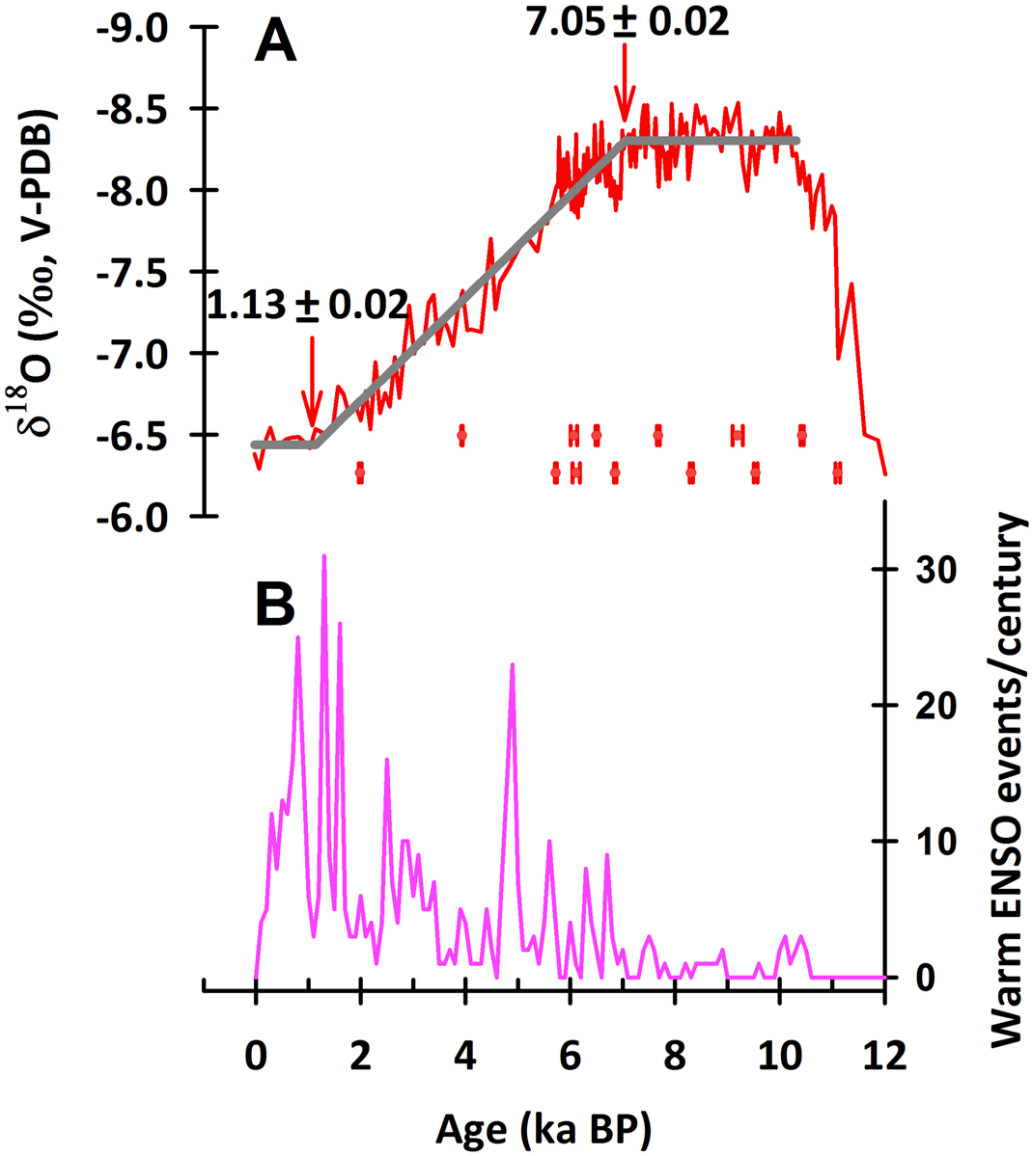
Proxy records of the FR5-inferred ASM and sediment-inferred ENSO activities over the past 12 kyrs. (**A**) Stalagmite FR5-inferred ASM variation. The gray line shows the RAMPFIT^25^ results with two inflection points, 7.05 ± 0.02 and 1.13 ± 0.02 ka BP. (**B**) Lake Laguna Pallcacocha sediment-inferred ENSO activity^41^.

The degradation of regional ASM conditions during the Holocene was reconstructed^27^. The RAMPFIT-determined transitions from the Holocene optimum to the Middle-Holocene degradation occur at 7.31 ± 0.10, 7.01 ± 0.06, 6.64 ± 0.03, 5.74 ± 0.06 and 5.76 ± 0.06 ka BP, according to the stalagmite records from the Qunf cave^26^, Dongge cave^37^, Shigao cave^27^, Heshang cave^38^ and Sanbao cave, respectively (Fig. 4 of ref.^27^). This transition in the FR5 record is determined to be 7.05 ± 0.02 ka BP (Fig. 6A), which is consistent with the timing of the Dongge record, but differs from the others. Our record supports the previous observations of the HOP termination being regionally asynchronous over the AM area^27^. In southern and eastern China, the summer monsoon precipitation is influenced by both the ISM and the East Asian summer monsoon (EASM). Jiang et al.^27^ proposed that the asynchronies could be attributed to sea surface temperature (SST) changes in the western tropical Pacific, the primary moisture source for the East Asian monsoon.

**Figure 7:**
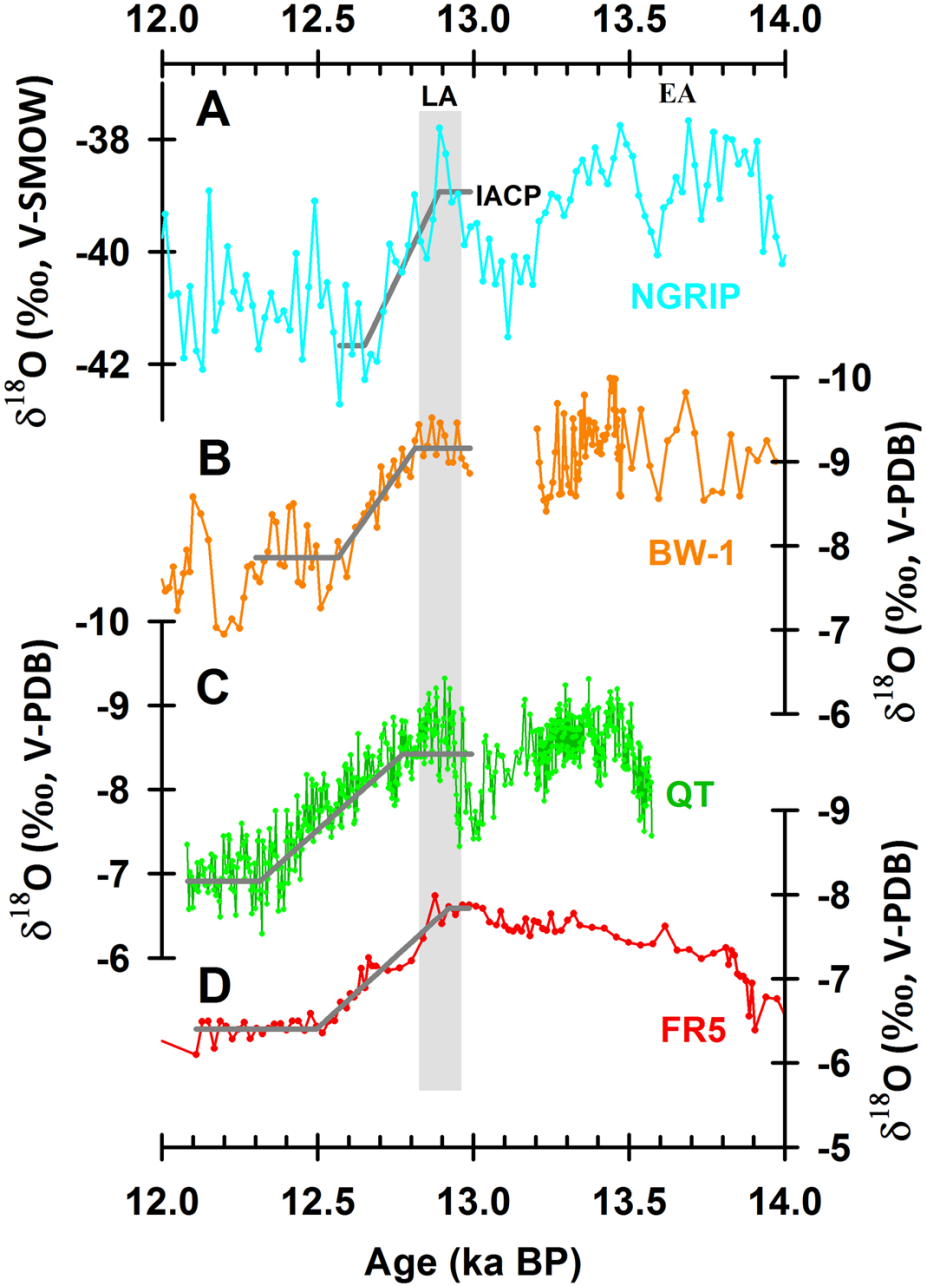
Comparison of the transition from the Allerød to the Younger Dryas recorded by different proxy records, including the δ^18^O records from (**A**) the Greenland ice core NGRIP^46^, (**B**) the Kulishu stalagmite BW-1^49^, (**C**) the Qingtian stalagmite QT^48^, and (**D**) the Furong stalagmite FR5 (this study). LA, IACP, EA represent the Late Allerød, intra-Allerød Cold Period, and Early Allerød, respectively. The gray lines represent the best fits from RAMPFIT^25^.

A recent study by Zhao et al.^39^ proposed that the millennial-scale change of the EASM intensity strongly correlated with the El Niño-Southern Oscillation (ENSO) during the late Holocene. Ta^40^ argued that an SST gradient from east to west in the tropical Pacific modulated the ENSO-like state and affected climate change in the monsoon region of China (Fig. 7 of ^40^). The gradual weakening of the ASM from the middle to late Holocene observed in the FR5 records matches the enhancement of the El Niño activities (Fig. 6) inferred from the sedimentary laminae of Lake Laguna Pallcacocha, southern Ecuador^41^. Although there are some arguments about the reliability of Pallcacocha records^42^, recent studies based on instrumental observations revealed a positive isotope-El Niño Southern Oscillation (ENSO) response^43^. Wang et al.^44^ also argued the impact of the ENSO on precipitation δ^18^O in East Asian monsoon by changing the convection and precipitation in the monsoon upstream regions. Their quantitatively calculation indicates that the contribution of ENSO accounts for about 3.1‰ in the 6‰ variation of Chinese stalagmite δ^18^O on the orbital timescale^44^. The FR5 record supports the argument that enhanced ENSO activity could result in the eastward migration of a weakened Walker circulation to the eastern Pacific. The induced westward movement of a strengthened Northwest Pacific subtropical high towards southwest China enhanced the proportion of Pacific monsoonal precipitation in eastern and southern China, absorbing more of the local vapor transport and resulting in heavy precipitation with high δ^18^O^35,39,40^. In consideration of the remarkable inconsistencies existing in centennial- to millennial-long ENSO reconstructions (reviewed by ref. ^42^), the reliable investigation about the correlation between ASM precipitation δ^18^O and ENSO should be based on more robust ENSO reconstructions and modelling to further understand the physical mechanisms.

### Younger Dryas, Older Dryas, and Bølling/Allerød

**Figure 8:**
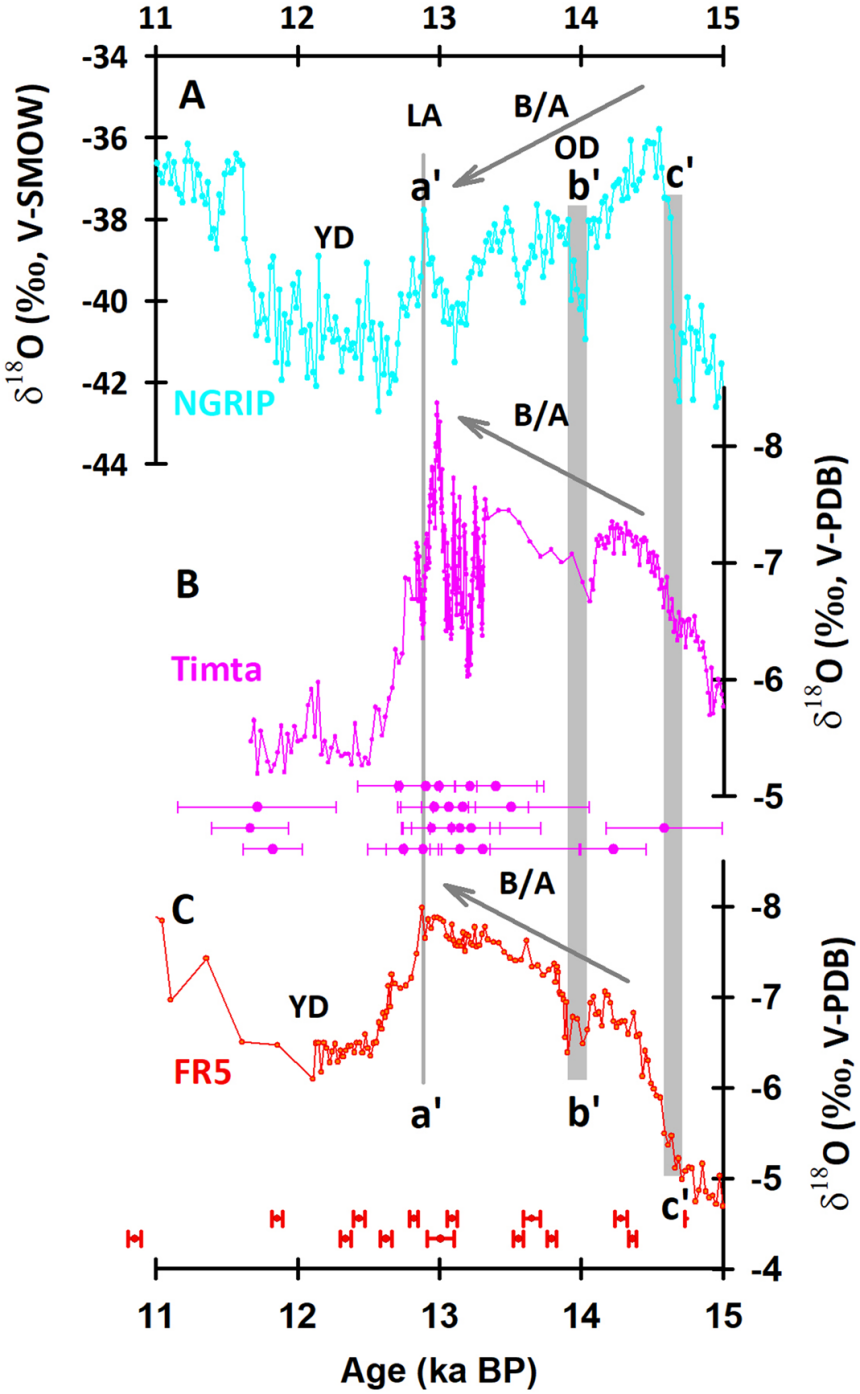
Comparison of δ^18^O records between (**A**) the Greenland NGRIP ice^46^ (**B**) Stalagmites from Timta Cave, India (Sinha et al., 2005), and (**C**) the stalagmite FR5 from 15 to 11 ka BP. YD, LA, B/A, OD denote the Younger Dryas, Late Allerød, Bølling-Allerød, and Older Dryas, respectively. The gray bars labelled a′, b′, and **c′** show the transition from B/A to YD, OD, and the transition from the Last Glacial period to B/A, respectively.

The YD, the most remarkable cold reversion of the climate after the termination of the LGP, was proposed to be caused by a slowdown or reduction of the AMOC and the associated abrupt increase of fresh melt water into the North Atlantic Ocean^45^. The YD was also characterized by a notable decrease of air temperature over central Greenland^28^. Due to the unprecedented high resolution and precise chronology of FR5 record, the details of a series of climate events can be refined accurately. Comprehensive comparison will be conducive to probe into the mechanism of interactions between climatic systems under different background. The onset of the YD as recorded in the FR5 δ^18^O record began at 12.92 ± 0.04 ka BP and ended at 12.50 ± 0.04 ka BP (Figs. 7D, 8C), followed by a weak millennial ASM interval reaching a δ^18^O maxima of -6.09‰ at 12.11 ± 0.04 ka BP. The beginning of the YD onset in the FR5 records closely matches that at 12.89 ± 0.14 ka BP in the NGRIP record and 13.0 ± 0.15 ka BP in the Timta Cave record, India (Fig. 7A, and a’ in Fig. 8)^46,47^.

The onset of the transition into the YD lasted for 450 ± 140 years in central China (Fig. 7C)^48^, which is consistent with the 420 ± 60 years observed in Furong Cave. A shorter transition of 240 ± 60 years is recorded in northern China (Fig. 7B)^49^, and a more gradual transition of 570 ± 110 years is observed in Philippines in the western tropical Pacific^11^. This meridional difference suggests regional hydroclimatic responses to the southward-moving NH high-latitude forcing that originates in the North Atlantic^10,11^.

The short centennial OD event, triggered by the slowdown of the AMOC during the Bølling/Allerød (B/A)^6^, began from 13.87 ± 0.05 to 14.06 ± 0.04 ka BP based on three ^230^Th dates in the FR5 record and lasted for 190 ± 60 years, which concurs with those dates noted in the Greenland δ^18^O and Indian stalagmite records which reflected the variation of Indian summer monsoon (ISM) (b’ in Fig. 8)^46,47,50^. These agreements indicate an instantaneous influence of the NH high-latitude forcing on the ASM through a reorganization of atmospheric circulations^28,51^.

**Figure 9:**
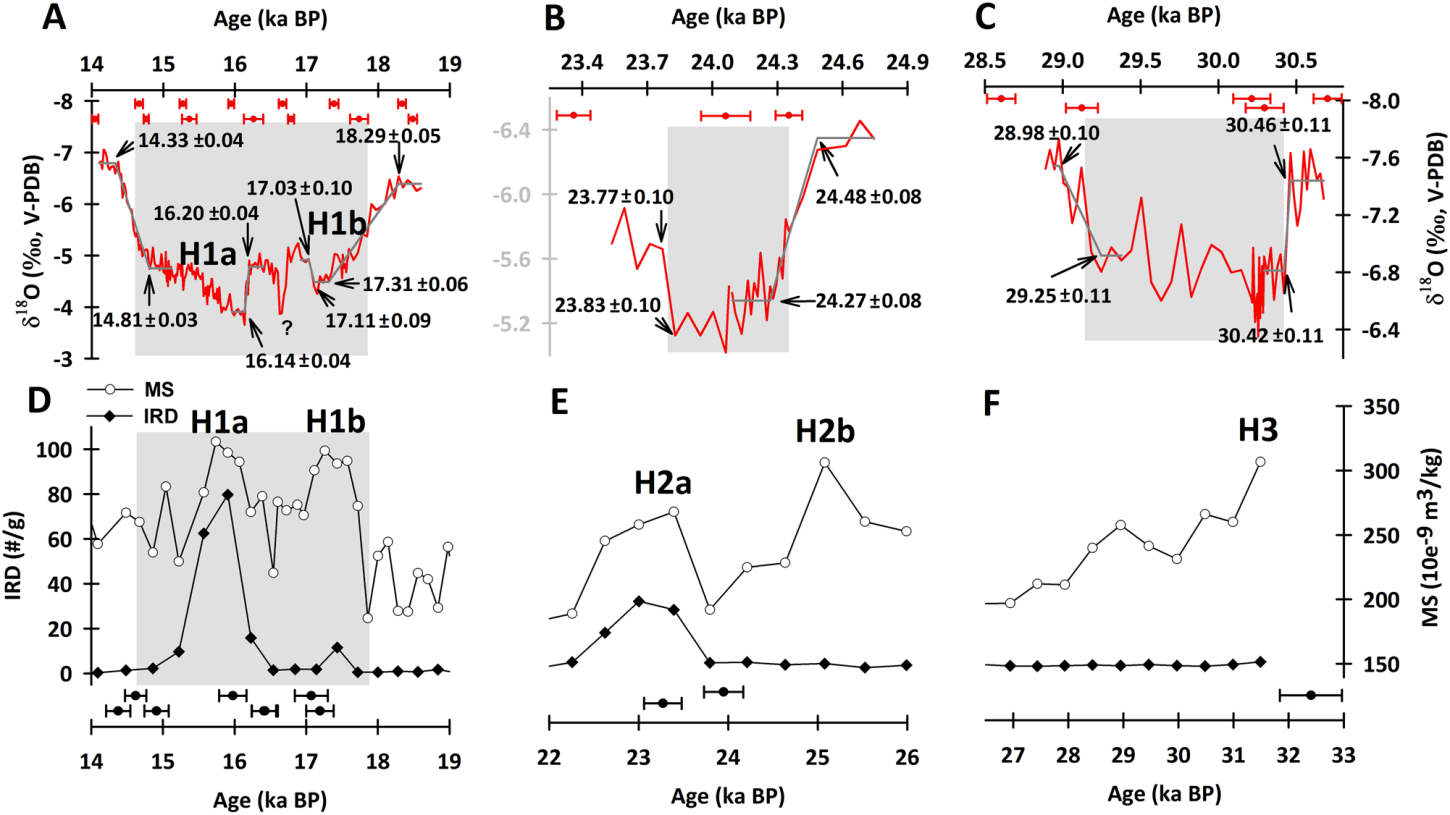
Stalagmite and marine records for H1, H2, and H3. (**A–C**) Furong δ^18^O records (red) with the best fits from the RAMPFIT program^25^. ^230^Th ages and errors are given by record. (**D–F**) The records of the ice-rafted detritus (IRD) (black lines with solid diamonds) and magnetic susceptibility (MS) (gray lines with hollow circles) are derived from marine sediment core SU8118 in the North Atlantic^1^. ^14^C ages with their errors (in black) are given by record.

In the FR5 record, the transition to B/A, lasting from 14.81 ± 0.03 to 14.33 ± 0.04 ka BP, is centered at 14.57 ka BP and lasted 480 ± 50 years (Figs. 8, 9A). However, the gradual multicentennial enhancement of the ASM differs from the abrupt 40 year warming in Greenland (c′ in Fig. 8A)^46^, but consistent to the gradually strengthen of ISM (c′ in Fig. 8B)^47^. These slow low-latitude hydroclimatic responses could require a long adjustment time to the dominant hemispheric-scale thermal forcing from Greenland^11^. In addition, the consistency between the NGRIP and FR5 records during the period of YD and B/A, (a′, b′ and c′ in Fig. 8) validates the accuracy of GICC05 chronology (Table 1).

During the B/A period, the NGRIP δ^18^O record shows a gradual millennial temperature decline, while the FR5 record expresses an increasing ASM intensity trend (Fig. 8). This discrepancy is also observed in other Chinese records from the caves of Hulu^9^ and Dongge^30^, as well as in Japanese records from Maboroshi Cave^52^, and could be attributed to the rising NHSI (Fig. 4B)^49^ or the enhanced cross-equatorial transport of moisture and heat from the southern oceans^52^.

### Heinrich events

H events are identified by the massive surges and melts of icebergs in the North Atlantic, perturbed the global ocean circulation and triggered a slowdown of the AMOC^1,2,12^. Stalagmite records show that the ASM intensity could be weakened during H events through atmospheric teleconnections^9^. The 37-kyr FR5 δ^18^O record demonstrates weak ASMs during H1, H2, and H3, which are centered at 16.2, 24.0, and 29.8 ka BP, respectively (Figs. 4, 9).

The onset of a weak ASM, accompanied by an increase of 2.5‰ in the δ^18^O during H1, spanned from 18.29 to 17.31 ka BP and lasted 0.98 kyrs. The recovery, spanning from 14.81 to 14.33 ka BP, lasted 0.48 kyrs (Fig. 9A). The maximum δ^18^O value of -3.65‰ is observed at 16.14 ka BP. This interval can be divided into two phases, 16.20–14.33 and 18.29–17.03 ka BP (Fig. 9A), corresponding to the substages of H1a and H1b in the North Atlantic sediment records (Fig. 9D). This double-peak event is also detected in the South American monsoon system, where it is called the “Maga-South Atlantic Convergence Zone (SACZ)”^53^. The evidence again indicates that this high-latitude forcing affected inter-hemispheric hydroclimates. The 1.26-kyr H1b is characterized by a slow 1-kyr onset and a fast 80-yr ending. While the onset of the 1.87-kyr H1a lasted only 60 years, the termination took 480 years (Fig. 9A). A 0.3-kyr weak ASM, registered by the FR5 record at 16.63 ka BP, has not yet been observed in any North Atlantic records (Fig. 9A), but could be a regional ASM event or limited by the intrinsic millennial resolutions of high-latitude marine records.

During H2, FR5 indicates the onset of a weak ASM, with an enrichment of 1.2‰ in δ^18^O from 24.48 to 24.27 ka BP; the recovery spanned from 23.83 to 23.77 ka BP (Fig. 9B). This single-peak event is consistent with evidence of IRD events and alkenone-inferred salinity changes recorded in the North Atlantic sediment core SU8118^1^. The corresponding weak ASM of H3 shows an onset transition of 1‰ in the FR5 δ^18^O record, lasting from 30.46 to 30.42 ka BP, and was only 40 years long. In contrast, the termination of H3, from 29.25 to 28.98 ka BP, lasted 270 years (Fig. 9C).

The abrupt onsets at H3 (40 years) and H1a (60 years) and the terminations at H2 (60 years) and H1b (80 years) in the FR5 record (Fig. 9) are not unique in the monsoonal region. The stalagmite M1-2 δ^18^O record from Moomi Cave, Socotra Island, Yemen, shows a 25-yr recovery of the ISM intensity at the end of H5^54^. The observed abrupt hydrological change of the Indian monsoon could be attributed to the fast meridional migration of the ITCZ responding to the recovery of the AMOC via atmospheric reorganization^54^.

The Furong record also clearly shows different transitional durations (TDs) of the onsets and terminations of the weak ASM periods of H1–3 (Fig. 9). The TDs of the onsets/terminations are 980/480, 210/60, and 40/270 years for the weak ASMs of H1, H2 and H3, respectively (Fig. 9). The relatively slow onsets and fast terminations of H1 and H2 are opposite those of H3. This discrepancy cannot be attributed to the global sea level change (Fig. 4), but the exact mechanism causing these inconsistent ASM responses between the different H events is not clear. We speculate that the composition of the orbitally controlled changes in insolation and the rapid changes in the oceanic and atmospheric circulation patterns during the NH cooling events control the different responses of the ASM. The strength of the ASM may be particularly sensitive to NH cooling when the continent is at its warmest due to a precessional insolation maximum (Fig. 4B), such that H3 is characterized by an abrupt weakening of the ASM (Fig. 9C). More comprehensive models and simulations are necessary to fully verify the physical mechanism of this hypothesis.

### Decoupling between high and mid-low latitudes

**Figure 10:**
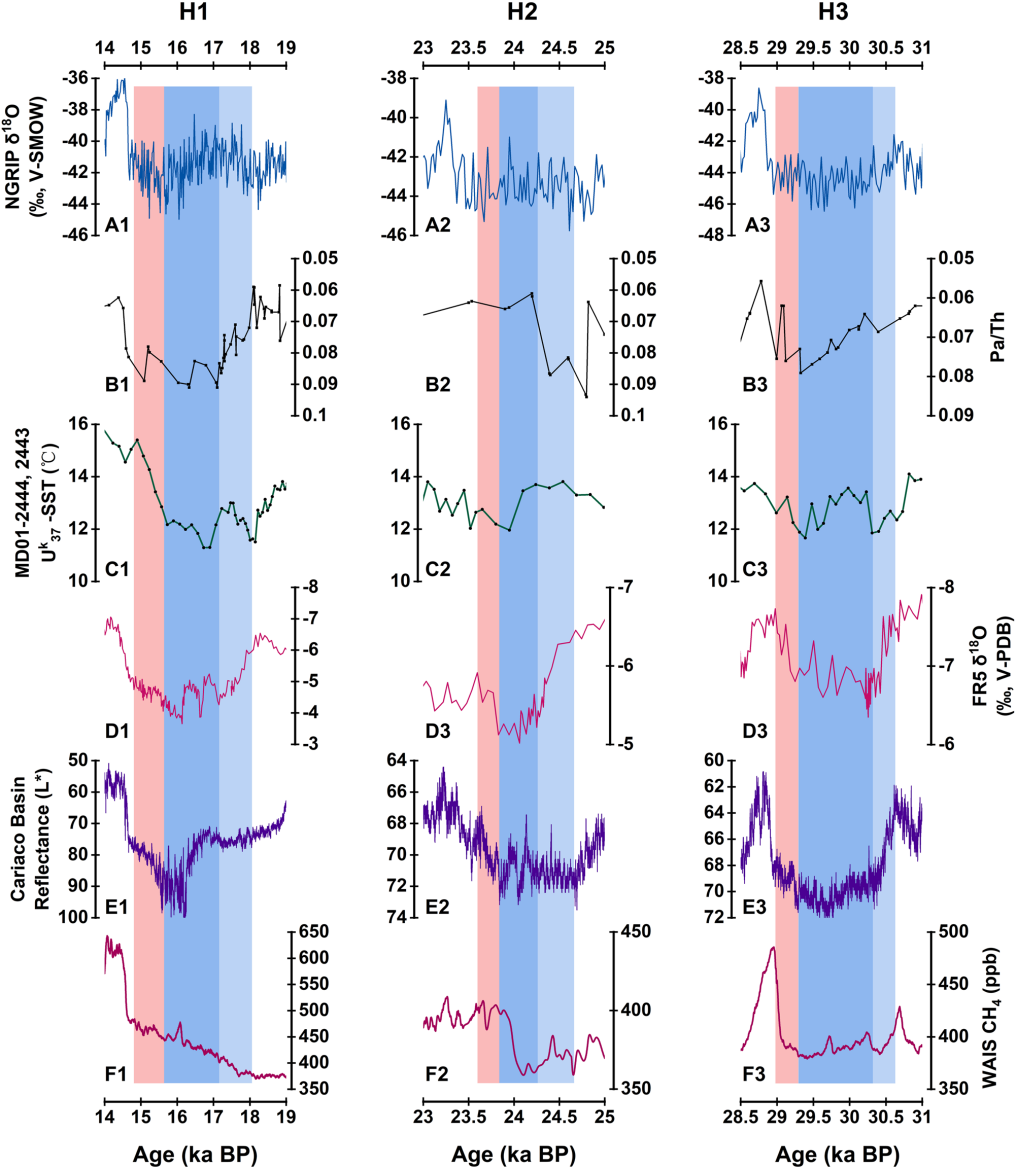
Comparison of paleoclimate records of H1-3 in different latitudes. (**A**) δ^18^O record of the NGRIP ice core^21,55^. (**B**) North Atlantic ocean sediment cores compilation Pa/Th record^67^. (**C**) Sea surface temperature records reconstructed from Iberian margin marine sediment cores MD01-2444 and MD01-2443^68^. (**D**) δ^18^O record of stalagmite FR5 (this study). (**E**) Reflectance records of Cariaco Basin sediment cores MD03-2621^57^. (**F**) CH_4_ record in the WAIS ice core^60^. The light blue and orange bands indicate the onset and termination of H events, respectively.

There is obvious decoupling between the climate change in NH high latitudes and low latitude during H1, H2 and H3 (Fig. 10). The comparison of FR5 records with other paleoclimate records indicates that the Greenland ice core δ^18^O remains stable during H1, H2 and H3, which means that Greenland was in a state of relative stable low-temperature (Fig. 10A1–A3) (NGRIP^21^. However, this stable state is almost not observed in other climate records in different latitudes, including FR5. Specifically, there is no abrupt change in Greenland temperature at 18.29, 24.48 and 30.46 ka BP, the start of H 1–3 (Fig. 10A)^21,55^. While, the abrupt decline of ASM (increase of FR5 δ^18^O) at these times indicates that the low latitude climate systems related to ITCZ have undergone an intense change (Fig. 10D,E)^18,56,57^. On the other hand, the strengthening of ASM is several centuries prior to the warming of Greenland at the end of H 1–3 (Fig. 10A,D). The decoupling between the high and low latitude climate variations indicates that the climate change in other parts of the world during the period of H events cannot be completely attributed to the temperature change in the high latitude regions (e.g. Greenland)^15,17^.

Previous researches based on the comparison of ice cores and marine sediments indicate that the tropical atmospheric circulation in the period of H4 and last deglaciation is also decoupled from the temperature changes in Greenland^15,17^. Iceberg discharge from the Laurentide ice sheet occurred several centuries after the cooling of North Atlantic surface ocean and decrease of AMOC intensity^58,59^. During H4, southward shift of the ITCZ delayed by 550 ± 60 years after the beginning of the cold period in Greenland^15^. The abrupt increase of WAIS CH_4_ concentration also occurred under the condition of stable temperature in Greenland, suggesting that the position of ITCZ was shifting further south at this time (Fig. 10F)^60^. Constrained by annually laminate chronology, three different phases were identified in the low latitude stalagmite records from Brazil, distinct to the relatively stable low-temperature state during the H4 in Greenland^18^. Our FR5 stalagmite record further support the inference that large-scale reorganization of atmospheric water cycle occurred in the low latitudes during the period of H events, while the temperature in Greenland remained stable^15–17^. However, recent researches on YD and Dansgaard-Oeschger events (D/O) since the last glacial indicate practically synchronous climate changes in high to low latitudes during the onsets of these abrupt interstadial events^61^. In addition, the onset of YD in the North Atlantic, Greenland, and the Asian Monsoon region are synchronous within a few decades (Fig. 8)^62^. All the above mentioned observations suggest possible different mechanisms for the correlation between high and mid-low latitudes climate change at the different background of H events, and YD, D/O events. This supposition argued the previous speculation which proposed that series centurial- millennial abrupt climatic events at low latitude during the LGP were dominated by the climate changes in high latitude. Our high quality records suggest possible different correlation between the high and mid-low latitudes under glacial and inter-glacial background. For example, in the late Holocene, at ~ 2 ka BP, the change of ASM could be modulated by the change in AMOC (Cheng et al.^63^) or greenhouse gas forcing, which offsets summer insolation forcing^64^. More precise chronology and high-resolution constrained records and model simulations are essential to address this supposition.

## Conclusions

We established an accurate chronology for the stalagmite-inferred evolution of the ASM over the past 37 ka from Furong Cave, southwestern China. The weak ASM events of the YD, OD, and H1–3, are precisely determined, with age uncertainties of few decades. Our record shows surprisingly different temporal responses of the monsoonal hydroclimate during the transitions of the onsets and recoveries of the weakened ASM of the first three H events. During the period of H1–3, the observed decoupling between the climate changes in the mid-low latitudes and Greenland indicates that the change of atmospheric water cycle in low latitudes was generated by the southward shift of ITCZ. On the other hand, under the deglacial boundary conditions, the onset of YD, OD and BA in the Northern high latitudes and the Asian Monsoon region are synchronous within a few decades (Fig. 8). There is possible different correlation between the high and mid-low latitudes under glacial and inter-glacial background. These observations argue the traditional opinion about the correlation between the climate change in high latitude and mid-low latitudes. The change of ASM could not be always triggered by the northern high latitudes. As the major energy and vapor source of ASM, the topical Indian Ocean and Pacific Ocean maybe play a crucial role in modulate the evolution of ASM, especially under the present background of global warming.

## Supplementary Information

Supplementary Information.
