# Supplementary material for: High precise dating on the variation of the Asian summer monsoon since 37 ka BP
Source: Sci Rep. 2021 Apr 30;11:9375. doi: 10.1038/s41598-021-88597-7 (PMC8087833; doi:10.1038/s41598-021-88597-7)
Supplement: Supplementary file 1 — Supplementary Information. [file 41598_2021_88597_MOESM1_ESM.doc]

# Supplementary materials

# High precise dating on the variation of the Asian summer monsoon since 37 ka BP

Ting-Yong Li ^a^ *, Yao Wu ^b^, Chuan-Chou Shen ^c^ *, Jun-Yun Li ^b^, Hong-Wei Chiang ^c^, Ke Lin ^c, §^, Liang-Cheng Tan ^d^, Xiu-Yang Jiang ^e^, Hai Cheng ^f, g^, R. Lawrence Edwards ^g^

^a^ *Yunnan Key Laboratory of Plateau Geographical Processes and Environmental Change, Faculty of Geography, Yunnan Normal University, Kunming 650500, China*

^b^ *Chongqing Key Laboratory of Karst Environment,* *School of Geographical Sciences; Southwest University, Chongqing 400715, China*

^c^ *High-Precision Mass Spectrometry and Environment Change Laboratory (HISPEC), Department of Geosciences, National Taiwan University, Taipei, 10617, Taiwan*

*^d^ State Key Laboratory of Loess and Quaternary Geology, Institute of Earth Environment, Chinese Academy of Sciences, Xi'an 710075, China*

^e^ *College of Geographical Science; Fujian Normal University, Fuzhou 350007, China*

*^f^* *Institute of Global Environmental Change, Xi'an Jiaotong University, Xi'an 710049, China*

*^g^* *Department of Earth Sciences, University of Minnesota, Minneapolis, MN 55455, USA*

**^§^** The present affiliation of Lin K. is the [Earth Observatory of Singapore](https://www.researchgate.net/institution/Nanyang_Technological_University/department/Earth_Observatory_of_Singapore), [Nanyang Technological University,](https://www.researchgate.net/institution/Nanyang_Technological_University) 639798, Singapore.

**Appendix Table 1.** Uranium-series dating results for stalagmite FR5, dated in the high-precision mass spectrometry and environment change laboratory (HISPEC) of National Taiwan University

| Depth (mm) | ^238^U | | ^232^Th | | *δ *^234^U | | [^230^Th/^238^U] | | [^230^Th/^232^Th] | | Age (ka) | | Age (ka BP) | | *δ* ^234^U_initial_ | |
| --- | --- | --- | --- | --- | --- | --- | --- | --- | --- | --- | --- | --- | --- | --- | --- | --- |
|  | (ppb) | | (ppt) | | measured ^a^ | | activity ^c^ | | (x 10^-6^ ^d^) | | uncorrected | | corrected ^c,e^ | | corrected ^b^ | |
| 9.5 | 296.8 | ±0.2 | 331.9 | ±18.8 | 3448.8 | ±3.8 | 0.0830 | ±0.0011 | 1226 | ±71 | 2.056 | ±0.028 | 1.991 | ±0.028 | 3462.0 | ± 3.8 |
| 20 | 794.7 | ±0.7 | 2056.0 | ±7.6 | 3649.6 | ±3.2 | 0.1680 | ±0.0006 | 1072 | ±6 | 4.007 | ±0.016 | 3.935 | ±0.017 | 3683.8 | ± 3.2 |
| 30 | 830.5 | ±0.8 | 8.2 | ±6.8 | 3675.7 | ±5.7 | 0.2425 | ±0.0006 | 404784 | ±337336 | 5.786 | ±0.016 | 5.727 | ±0.016 | 3729.0 | ± 5.7 |
| 40 | 547.6 | ±1.7 | 352.9 | ±8.2 | 3628.5 | ±16.8 | 0.2541 | ±0.0022 | 6510 | ±159 | 6.133 | ±0.059 | 6.070 | ±0.059 | 3684.6 | ± 17.1 |
| 48.5 | 767.1 | ±2.9 | 290.2 | ±6.8 | 3668.3 | ±20.7 | 0.2580 | ±0.0027 | 11260 | ±286 | 6.175 | ±0.073 | 6.114 | ±0.073 | 3725.6 | ± 21.1 |
| 57 | 486.9 | ±0.5 | 1270.2 | ±4.3 | 3704.0 | ±5.1 | 0.2765 | ±0.0009 | 1750 | ±8 | 6.576 | ±0.023 | 6.505 | ±0.024 | 3765.9 | ± 5.2 |
| 67.5 | 412.0 | ±0.4 | 11.2 | ±6.4 | 3847.2 | ±5.4 | 0.2993 | ±0.0008 | 182418 | ±104279 | 6.916 | ±0.020 | 6.857 | ±0.020 | 3915.5 | ± 5.5 |
| 80.5 | 499.6 | ±0.5 | 852.0 | ±3.9 | 4192.1 | ±4.8 | 0.3581 | ±0.0011 | 3466 | ±19 | 7.745 | ±0.026 | 7.679 | ±0.026 | 4276.6 | ± 4.9 |
| 89.5 | 421.9 | ±0.2 | 24.7 | ±16.3 | 4096.0 | ±3.2 | 0.3790 | ±0.0013 | 106908 | ±70589 | 8.370 | ±0.029 | 8.312 | ±0.029 | 4185.9 | ± 3.3 |
| 95 | 307.0 | ±1.6 | 354.0 | ±5.9 | 4246.3 | ±28.1 | 0.4304 | ±0.0037 | 6163 | ±111 | 9.262 | ±0.096 | 9.197 | ±0.096 | 4350.5 | ± 28.8 |
| 97 | 271.3 | ±0.3 | 11.9 | ±4.6 | 4138.1 | ±5.9 | 0.4364 | ±0.0013 | 164740 | ±63949 | 9.600 | ±0.032 | 9.540 | ±0.032 | 4243.7 | ± 6.1 |
| 104 | 771.3 | ±0.9 | 12.0 | ±5.6 | 3782.4 | ±6.2 | 0.4422 | ±0.0010 | 470337 | ±218041 | 10.485 | ±0.029 | 10.426 | ±0.029 | 3888.5 | ± 6.4 |
| 109.5 | 988.4 | ±2.0 | 77.8 | ±13.4 | 3808.4 | ±11.8 | 0.4724 | ±0.0015 | 99096 | ±17123 | 11.165 | ±0.046 | 11.107 | ±0.046 | 3922.7 | ± 12.1 |
| 111.5 | 1072.4 | ±1.4 | 484.4 | ±3.2 | 3528.1 | ±5.3 | 0.4831 | ±0.0013 | 17657 | ±122 | 12.169 | ±0.037 | 12.110 | ±0.037 | 3644.2 | ± 5.5 |
| 124 | 1213.1 | ±1.5 | 14.3 | ±5.0 | 3768.9 | ±7.1 | 0.5282 | ±0.0012 | 739161 | ±259768 | 12.652 | ±0.036 | 12.593 | ±0.036 | 3898.3 | ± 7.4 |
| 128 | 1836.3 | ±2.4 | 819.5 | ±3.9 | 3731.8 | ±6.0 | 0.5278 | ±0.0014 | 19527 | ±103 | 12.746 | ±0.040 | 12.687 | ±0.040 | 3860.9 | ± 6.2 |
| 130.5 | 1200.9 | ±1.7 | 48.8 | ±13.5 | 3789.0 | ±7.7 | 0.5418 | ±0.0014 | 220054 | ±60820 | 12.935 | ±0.042 | 12.877 | ±0.042 | 3922.3 | ± 8.0 |
| 135 | 2793.0 | ±2.6 | 20.3 | ±4.9 | 3753.9 | ±5.5 | 0.5456 | ±0.0011 | 1237803 | ±297700 | 13.133 | ±0.031 | 13.074 | ±0.031 | 3888.1 | ± 5.7 |
| 142 | 1835.8 | ±6.9 | 241.3 | ±5.3 | 3606.6 | ±20.4 | 0.5359 | ±0.0028 | 67327 | ±1508 | 13.321 | ±0.095 | 13.262 | ±0.095 | 3737.5 | ± 21.2 |
| 144 | 1436.3 | ±1.4 | 106.4 | ±5.2 | 3665.8 | ±5.4 | 0.5459 | ±0.0012 | 121678 | ±5972 | 13.402 | ±0.034 | 13.342 | ±0.034 | 3799.7 | ± 5.6 |
| 150 | 2743.9 | ±2.9 | 65.4 | ±4.9 | 3619.4 | ±5.8 | 0.5585 | ±0.0012 | 387011 | ±29018 | 13.870 | ±0.037 | 13.810 | ±0.037 | 3756.6 | ± 6.0 |
| 155 | 3100.3 | ±6.7 | 82.4 | ±3.9 | 3547.2 | ±12.1 | 0.5532 | ±0.0017 | 343694 | ±16422 | 13.963 | ±0.059 | 13.904 | ±0.059 | 3682.5 | ± 12.5 |
| 157 | 2420.1 | ±2.2 | 46.7 | ±4.9 | 3475.8 | ±5.3 | 0.5497 | ±0.0011 | 470189 | ±48872 | 14.104 | ±0.035 | 14.045 | ±0.035 | 3609.8 | ± 5.5 |
| 169 | 5091.2 | ±6.8 | 838.8 | ±3.6 | 3488.5 | ±5.7 | 0.5694 | ±0.0014 | 57059 | ±274 | 14.591 | ±0.043 | 14.533 | ±0.043 | 3628.0 | ± 5.9 |
| 170.5 | 5231.6 | ±3.8 | 44.6 | ±12.6 | 3489.1 | ±3.6 | 0.5725 | ±0.0010 | 1108850 | ±313580 | 14.672 | ±0.029 | 14.614 | ±0.029 | 3629.4 | ± 3.7 |
| 179 | 4350.9 | ±4.9 | 47.3 | ±5.5 | 3550.0 | ±5.7 | 0.5956 | ±0.0012 | 904021 | ±104227 | 15.082 | ±0.037 | 15.023 | ±0.037 | 3697.2 | ± 6.0 |
| 185 | 7201.1 | ±20.2 | 278.0 | ±6.1 | 3612.4 | ±14.7 | 0.6066 | ±0.0024 | 259394 | ±5744 | 15.154 | ±0.081 | 15.095 | ±0.081 | 3762.9 | ± 15.3 |
| 201.5 | 6627.6 | ±5.2 | 36.5 | ±11.3 | 3647.1 | ±4.3 | 0.6290 | ±0.0011 | 1883737 | ±582588 | 15.621 | ±0.032 | 15.563 | ±0.032 | 3804.1 | ± 4.6 |
| 214 | 6153.0 | ±22.5 | 257.9 | ±5.7 | 3614.8 | ±20.7 | 0.6341 | ±0.0030 | 249757 | ±5530 | 15.871 | ±0.109 | 15.812 | ±0.109 | 3773.1 | ± 21.7 |
| 219.5 | 6197.6 | ±7.0 | 65.5 | ±5.5 | 3606.7 | ±5.9 | 0.6451 | ±0.0013 | 1007801 | ±84049 | 16.194 | ±0.042 | 16.135 | ±0.042 | 3768.1 | ± 6.2 |
| 222.5 | 6065.8 | ±5.2 | 51.0 | ±11.9 | 3519.5 | ±4.2 | 0.6364 | ±0.0011 | 1249902 | ±292691 | 16.290 | ±0.033 | 16.232 | ±0.033 | 3677.9 | ± 4.4 |
| 231 | 7081.5 | ±9.0 | 187.2 | ±7.6 | 3423.9 | ±6.3 | 0.6402 | ±0.0015 | 399775 | ±16311 | 16.770 | ±0.049 | 16.711 | ±0.049 | 3582.8 | ± 6.6 |
| 235 | 8189.3 | ±24.0 | 845.9 | ±6.7 | 3451.0 | ±14.6 | 0.6543 | ±0.0029 | 104579 | ±898 | 17.051 | ±0.100 | 16.991 | ±0.100 | 3614.0 | ± 15.3 |
| 241 | 5072.8 | ±5.1 | 303.3 | ±6.9 | 3296.5 | ±5.1 | 0.6481 | ±0.0013 | 178968 | ±4067 | 17.531 | ±0.043 | 17.472 | ±0.043 | 3456.8 | ± 5.3 |
| 246 | 3775.2 | ±5.0 | 1464.0 | ±4.9 | 3126.5 | ±5.5 | 0.6263 | ±0.0014 | 26667 | ±100 | 17.650 | ±0.048 | 17.591 | ±0.048 | 3279.5 | ± 5.8 |
| 251 | 2703.9 | ±3.1 | 213.6 | ±7.1 | 3235.3 | ±5.7 | 0.6646 | ±0.0015 | 138921 | ±4621 | 18.283 | ±0.052 | 18.223 | ±0.052 | 3399.9 | ± 6.0 |
| 262 | 2628.0 | ±4.6 | 435.2 | ±5.0 | 3477.7 | ±10.4 | 0.7520 | ±0.0017 | 74970 | ±876 | 19.654 | ±0.069 | 19.594 | ±0.069 | 3668.8 | ± 11.0 |
| 270.5 | 2965.2 | ±2.9 | 784.3 | ±3.4 | 3384.0 | ±4.0 | 0.7487 | ±0.0013 | 46740 | ±216 | 20.013 | ±0.042 | 19.954 | ±0.042 | 3573.6 | ± 4.2 |
| 275.5 | 2737.4 | ±4.6 | 1015.6 | ±5.5 | 3685.8 | ±10.5 | 0.8305 | ±0.0019 | 36959 | ±208 | 20.817 | ±0.073 | 20.756 | ±0.073 | 3901.2 | ± 11.2 |
| 283.5 | 1997.1 | ±3.1 | 208.7 | ±12.8 | 3720.8 | ±9.1 | 0.8731 | ±0.0023 | 137948 | ±8472 | 21.794 | ±0.077 | 21.735 | ±0.077 | 3949.2 | ± 9.8 |
| 295 | 2278.4 | ±2.7 | 679.8 | ±3.8 | 4120.4 | ±5.7 | 0.9882 | ±0.0019 | 54683 | ±319 | 22.808 | ±0.055 | 22.749 | ±0.055 | 4386.0 | ± 6.1 |
| 301.5 | 1619.8 | ±2.3 | 216.5 | ±13.6 | 3812.7 | ±8.1 | 0.9514 | ±0.0024 | 117509 | ±7406 | 23.420 | ±0.078 | 23.362 | ±0.078 | 4065.4 | ± 8.7 |
| 307.5 | 2400.6 | ±5.3 | 89.3 | ±5.6 | 3856.6 | ±15.6 | 0.9865 | ±0.0028 | 437978 | ±27489 | 24.121 | ±0.114 | 24.062 | ±0.114 | 4120.4 | ± 16.7 |
| 317.5 | 2991.6 | ±3.7 | 620.4 | ±3.8 | 4036.5 | ±6.2 | 1.0346 | ±0.0020 | 82376 | ±521 | 24.411 | ±0.061 | 24.353 | ±0.061 | 4316.1 | ± 6.7 |
| 324 | 3788.4 | ±11.3 | 15.2 | ±4.8 | 3984.9 | ±20.6 | 1.0566 | ±0.0039 | 4339137 | ±1362175 | 25.262 | ±0.153 | 25.203 | ±0.153 | 4271.2 | ± 22.1 |
| 336.5 | 2588.7 | ±3.7 | 60.6 | ±12.8 | 4025.3 | ±8.2 | 1.0872 | ±0.0027 | 767259 | ±161841 | 25.831 | ±0.084 | 25.773 | ±0.084 | 4321.4 | ± 8.9 |
| 344 | 2389.0 | ±3.2 | 66.3 | ±4.9 | 4090.1 | ±9.0 | 1.1276 | ±0.0022 | 671339 | ±49699 | 26.509 | ±0.077 | 26.450 | ±0.077 | 4399.5 | ± 9.8 |
| 356 | 2872.9 | ±3.4 | 539.8 | ±2.5 | 4101.8 | ±6.2 | 1.1424 | ±0.0022 | 100378 | ±488 | 26.821 | ±0.068 | 26.763 | ±0.068 | 4415.9 | ± 6.7 |
| 365 | 2121.0 | ±3.9 | 18.9 | ±5.9 | 4145.5 | ±14.3 | 1.1737 | ±0.0030 | 2177933 | ±683745 | 27.371 | ±0.113 | 27.312 | ±0.113 | 4469.9 | ± 15.5 |
| 374.5 | 2101.9 | ±3.0 | 68.0 | ±12.5 | 4437.3 | ±8.7 | 1.2600 | ±0.0031 | 643473 | ±117939 | 27.839 | ±0.090 | 27.781 | ±0.090 | 4791.0 | ± 9.4 |
| 381.5 | 2303.6 | ±1.4 | 116.2 | ±13.8 | 4366.7 | ±4.2 | 1.2646 | ±0.0021 | 413896 | ±49202 | 28.360 | ±0.058 | 28.302 | ±0.058 | 4721.7 | ± 4.6 |
| 392.5 | 1888.1 | ±2.6 | 57.4 | ±12.0 | 4134.0 | ±8.0 | 1.2209 | ±0.0029 | 662689 | ±138027 | 28.659 | ±0.091 | 28.601 | ±0.091 | 4473.8 | ± 8.7 |
| 401.5 | 1310.9 | ±2.1 | 59.0 | ±13.4 | 4212.8 | ±10.1 | 1.2600 | ±0.0032 | 462366 | ±104744 | 29.177 | ±0.103 | 29.119 | ±0.103 | 4565.7 | ± 11.1 |
| 410 | 2025.9 | ±3.8 | 289.8 | ±4.8 | 4239.4 | ±13.2 | 1.3092 | ±0.0032 | 151094 | ±2535 | 30.270 | ±0.119 | 30.211 | ±0.119 | 4608.8 | ± 14.5 |
| 416.5 | 1970.6 | ±3.5 | 16.6 | ±6.0 | 4193.5 | ±14.4 | 1.3008 | ±0.0030 | 2552328 | ±922117 | 30.352 | ±0.122 | 30.293 | ±0.122 | 4559.9 | ± 15.7 |
| 426 | 1496.3 | ±1.6 | 888.7 | ±4.5 | 4218.2 | ±6.0 | 1.3227 | ±0.0031 | 36772 | ±202 | 30.757 | ±0.090 | 30.697 | ±0.090 | 4592.0 | ± 6.6 |
| 435 | 1460.9 | ±3.0 | 251.7 | ±4.3 | 4219.9 | ±14.0 | 1.3639 | ±0.0045 | 130707 | ±2253 | 31.817 | ±0.152 | 31.757 | ±0.152 | 4607.6 | ± 15.5 |
| 440 | 1377.8 | ±2.7 | 280.1 | ±6.7 | 4279.3 | ±14.7 | 1.4236 | ±0.0035 | 115620 | ±2765 | 32.955 | ±0.138 | 32.895 | ±0.138 | 4687.5 | ± 16.2 |
| 448 | 1438.9 | ±2.6 | 104.1 | ±6.6 | 4484.3 | ±15.1 | 1.4826 | ±0.0036 | 338514 | ±21587 | 33.037 | ±0.137 | 32.977 | ±0.137 | 4913.4 | ± 16.6 |
| 451 | 1384.0 | ±1.4 | 573.7 | ±3.5 | 4400.6 | ±5.6 | 1.4624 | ±0.0027 | 58250 | ±366 | 33.103 | ±0.079 | 33.044 | ±0.079 | 4822.5 | ± 6.2 |
| 465.5 | 1600.9 | ±2.5 | 88.1 | ±5.9 | 4339.2 | ±13.4 | 1.4959 | ±0.0030 | 448703 | ±30004 | 34.401 | ±0.126 | 34.342 | ±0.126 | 4772.6 | ± 14.8 |
| 468 | 1570.9 | ±3.3 | 341.3 | ±4.8 | 4311.9 | ±13.4 | 1.5015 | ±0.0052 | 114112 | ±1647 | 34.750 | ±0.168 | 34.690 | ±0.168 | 4747.2 | ± 15.0 |
| 475 | 2175.3 | ±3.3 | 65.7 | ±6.2 | 4346.1 | ±12.9 | 1.5204 | ±0.0030 | 830707 | ±78288 | 34.985 | ±0.124 | 34.926 | ±0.124 | 4788.1 | ± 14.3 |
| 487 | 1051.6 | ±0.7 | 289.8 | ±19.1 | 4400.9 | ±4.5 | 1.5636 | ±0.0019 | 93691 | ±6189 | 35.696 | ±0.059 | 35.634 | ±0.059 | 4858.2 | ± 5.0 |
| 489 | 1674.7 | ±3.0 | 565.5 | ±6.0 | 4291.8 | ±13.6 | 1.5395 | ±0.0036 | 75282 | ±806 | 35.900 | ±0.143 | 35.839 | ±0.143 | 4740.4 | ± 15.1 |
| 492 | 1454.2 | ±1.2 | 305.3 | ±19.3 | 4287.2 | ±5.3 | 1.5616 | ±0.0020 | 122828 | ±7765 | 36.524 | ±0.067 | 36.463 | ±0.067 | 4743.7 | ± 5.9 |
| 497 | 1477.6 | ±1.5 | 586.2 | ±23.5 | 4117.6 | ±5.6 | 1.5284 | ±0.0021 | 63602 | ±2551 | 37.000 | ±0.075 | 36.938 | ±0.075 | 4562.1 | ± 6.3 |

^a^ *δ*^234^U = ([^234^U/^238^U]_activity_ - 1) x 1000.

^b^ *δ*^234^U _initial_ corrected was calculated based on ^230^Th age (T), i.e., *δ*^234^U _initial_ =*δ*^234^U _measured_ *X* e^λ234*T^, and T is corrected age.

^c^ [^230^Th/^238^U]_activity_ = 1 - e^-λ230T^ + (*δ*^234^U _measured_/1000)[λ_230_/(λ_230_ -λ_234_)](1 - e^-(λ230 -λ234) T^), where T is the age.

Decay constants are 9.1705 x 10^-6^ yr^-1^ for ^230^Th, 2.82206 x 10^-6^ yr^-1^ for ^234^U (Cheng et al., 2013a), and 1.55125 x 10^-10^ yr^-1^ for ^238^U (Jeffey et al., 1971).

^d^ The degree of detrital ^230^Th contamination is indicated by the [^230^Th/^232^Th] atomic ratio instead of the activity ratio.

^e^ Age corrections were calculated using an estimated atomic ^230^Th/^232^Th ratio of 4 ± 2 ppm.
